# Perfect Rainbow Tradeoff with Checkpoints Revisited

**DOI:** 10.1371/journal.pone.0166404

**Published:** 2016-11-17

**Authors:** Jin Hong

**Affiliations:** Department of Mathematical Sciences and ISaC, Seoul National University, Seoul 08826, Korea; West Virginia University, UNITED STATES

## Abstract

The rainbow tradeoff is an algorithm for inverting one-way functions that is widely used in practice to recover passwords from unsalted password hashes. An auxiliary technique referred to as checkpoints can be applied to the rainbow tradeoff to reduce the time taken for these inversions. Working out a rigorous theory that can explain and predict the effects of this technique involves delicate manipulations of the random function and is thus a challenging task. In this work, we compare three existing theoretical analyses of the checkpoint technique. We first demonstrate that the claims made by the three works are incompatible with each other. We then carry out experiments designed to highlight these incompatibilities, obtaining experimental evidences that show just one of the three analyses to be correct. Finally, we discuss the obscure theoretical errors made by the two inadequate analyses.

## Introduction

Time memory tradeoff [1] is a technique for inverting one-way functions and the rainbow tradeoff [2] is the most widely used such algorithm. There are commercially available softwares that can recover lost passwords, which were set to prevent unauthorized accesses to digital documents, and many of these rely on the cryptanalytic tradeoff technique to expedite the recovery. Law enforcement agencies are also known to be using tools based on cryptanalytic tradeoff algorithms.

Any time memory tradeoff algorithm consists of two separate phases. In the pre-computation phase, massive computations of the specific one-way function under consideration are carried out and a digest of the findings is stored as large tables. The online phase starts when the target image to be inverted is assigned. Further computations that reference the pre-computed tables are done to recover the input corresponding to the inversion target. For any specific one-way function, the pre-computation phase need only be carried out once, and the resulting tables may be used to invert any number of targets associated with the one-way function.

Although the tradeoff technique allows for the amount of online computations to be much smaller than that required by an exhaustive search of the input, the time taken by the online phase is still uncomfortably large for situations of practical interest, and a significant portion of this time is spent on dealing with what are referred to as false alarms. The deployment of checkpoints [3, 4] allows a portion of these false alarms to be dismissed without any extra computations, thus reducing the time taken to invert each target.

To implement the tradeoff technique in a manner that meets the intended user’s needs, one must be able to predict the behavior of the online phase algorithm running under a given set of system parameters. It is important to have an accurate theoretical analysis of the online phase algorithm, because the high cost of the pre-computation phase makes it impractical to choose the system parameters through a trial-and-error approach. An accurate analysis is difficult to obtain, as it must account for the effects of false alarms, and the task becomes even more complicated when one considers the use of checkpoints.

There are a few existing publications that analyze the effects of using checkpoints on the online time complexity of the (perfect table) rainbow tradeoff. The first of these were the article [3] and its extended version [4] that introduced the checkpoint technique. The arguments given in these two papers covered just the case when the rainbow tables were of a special type that are referred to as *maximal* perfect tables. The second analysis article [5] (which was written by the author of the current paper) treated the general perfect rainbow tables, but with the restriction that only a single checkpoint was used. An extension of this to the case of multiple checkpoints appeared in [6]. A more recent work [7] also contains a treatment of the general perfect rainbow tables with multiple checkpoints.

The first [3, 4] and the second [5] works overlap in that they both cover the case of maximal perfect tables with a single checkpoint, and one would expect the restriction of results from [5] to the special case of maximal perfect tables to match the results of [3, 4]. In this work, we will explicitly carry out this restriction and point out that the two results do not agree. We will also provide experimental data that conform to the claims of [5], but that are incompatible with the claims of [3, 4].

One would similarly expect the restriction of claims made by the third work [6] to the single checkpoint situation to match the claims made by the second work [5]. Once again, we will show that this is not the case and provide experimental evidence that supports the claims of [5] while challenging those of [6].

We will also provide careful discussions of where the mistakes were made by [3, 4] and [6]. Our findings and discussions should help researchers and practitioners from falling into the traps of similar very plausible, but erroneous, arguments.

## Short History of the Cryptanalytic Tradeoff Technique

The study of cryptanalytic time memory tradeoff methods began with the classical algorithm by Hellman [1]. This first algorithm was announced as an attack on blockciphers, but was clearly applicable to the inversion of any one-way function. Some early performance analyses and optimizations of this original method appeared in [8, 9].

According to [10], Rivest made the observation that the online time taken by Hellman’s algorithm could be reduced by using the notion of distinguished points (DP). Pre-computation phase of the original algorithm generated fixed-length chains through iterated applications of the one-way function, and the suggestion of Rivest was to iterate the one-way function for each chain until one arrived at an element satisfying a preset condition. Intuitively, one could expect the online phases of the modified and original algorithms to behave similarly, if the distinguishing property was set so that the average length of the chains ending at DPs was equal to the fixed length of the original algorithm chains, except that the DP method had the practical advantage of calling for much smaller number of table lookups. The modification also naturally brought about the concept of perfect tables. Unlike the original algorithm, with the DP method, one was assured of no duplicates within the pre-computation matrix, if just the endpoints of the chains were free of duplicates, and the removal of duplicates lead to higher efficiency and success rate of the online phase.

A study of the DP method performance that tried to take the non-uniform lengths of chains into account appeared in [11, 12], where [13] was cited as also having studied the DP method. Further advancements concerning the analysis of DP method performance were made by [14], and some its findings were repeated by [15].

After a long absence of improvements to the core algorithm, the rainbow table method [2] was announced, with the claim of it being advantageous over the DP method by a factor of at least two. This was soon followed by the auxiliary technique of checkpoints [3, 4], which allowed for some of the negative effects of false alarms on the online time to be reduced. Checkpoints are applicable to both the classical Hellman and rainbow methods. Although applying them to the DP method should also be possible, there are complications and this is not widely considered.

It is known [16, 17] that, in a certain sense, the three algorithms mentioned above, i.e., the classical Hellman, DP, and rainbow tradeoffs, all provide the best *asymptotic* performance one can hope for. However, full performance analyses of these three algorithms that are accurate enough for the purpose of comparing them against each other have become available only more recently. The accurate success probably of the classical Hellman tradeoff under general parameters was given by [18, 19]. Some details concerning the performance of rainbow tradeoff for the special case of maximal perfect tables were given by [3, 4], and much more details concerning the classical Hellman, perfect rainbow, and non-perfect rainbow tradeoffs appeared in [5]. These works [3–5] also discussed the algorithm performances under the deployment of checkpoints. The performance of the non-perfect DP tradeoff was analyzed accurately in [20] and the perfect DP tradeoff was treated by [21]. The works [20, 21] also gathered together the existing analyses and provided a comprehensive performance comparison of the three major algorithms in their perfect and non-perfect versions.

It is also worth mentioning that there is a subject closely related to the time memory tradeoff technique which is referred to as the time memory *data* tradeoff. The initial works [22, 23] in this direction combined a generic attack on streamciphers [24, 25] with the classical Hellman or DP tradeoff methods, and the resulting algorithm were applicable to any situation where the inversion of just one of multiple targets is meaningful. The attacks [22, 23] on streamciphers had practical implications, as they were eventually implemented [26, 27] in full. The straightforward multi-target adaptation of the rainbow method is known to perform worse [28] than the adaptations of the classical Hellman and DP tradeoffs, and the fuzzy rainbow tradeoff [16, 17] appeared later as the multi-target rainbow tradeoff variant of comparable performance.

## Notation and Conventions

We assume that the reader is familiar with the rainbow tradeoff technique and the checkpoint method. In particular, we assume knowledge of the following concepts: matrix stopping constant, reduction function, rainbow chain, pre-computation chain, starting point, ending point, online chain, pre-computation matrix, pre-computation table, removal of ending point collisions, perfect table, maximal perfect table, inversion target, merge of chains, false alarm, regeneration of the pre-computation chain to resolve an alarm, checkpoint, checkpoint column, checkpoint information. Note that the terms pre-computation *table* and pre-computation *matrix* refer to different concepts. The reader may find the beginning sections of [20] and [21] helpful in recalling these concepts.

Only the perfect table version of the rainbow tradeoff is relevant to this paper. The size of the search space is denoted by *N*. The pre-computation rainbow matrix is assumed to consist of *m* chains or rows. Each pre-computation chain is created to be of length *t*, so that a pre-computation matrix contains (*t* + 1) columns. The column of starting points is labeled the 0-th column and the ending point column is labeled the *t*-th column. Most of our discussions will focus on a single pre-computation table. The 1-st iteration of the online phase searches for the (reduced) inversion target among the ending points, so that the possibility of locating the correct answer to the inversion problem in the (*t* − 1)-th column of the pre-computation matrix is tested. We take the convention that an online chain starts from the unknown answer to the inversion target, so that the 1-st iteration of the online phase deals with an online chain of length 1, even though no computation of the one-way function is performed. Most of our discussions will assume the case where a single checkpoint of 1-bit information is used, and the position of this checkpoint will be named the *c*-th column.

During our calculations of equations, we will routinely hide approximations of multiplicative factor that is of $1+O(\frac{1}{t})$ order and write them as equalities. For realistic applications of the rainbow tradeoff, the parameter *t* will be large enough to make these approximations practically indistinguishable from equalities. One consequence of this approach is that the single alarm that leads to the correct answer need not be distinguished from the strictly false alarms. That is, when stating the probability for a certain class of false alarms, which we know to be of Θ(1) order, we can simply consider all alarms, since the single alarm that leads to the correct answer will add at most $O(\frac{1}{t})$ to the probability. The simplifications of many formulas obtained through these approximations make it easier to focus on the fundamental differences between the claims made by the three works under consideration.

Throughout this paper, the arguments and claims made by [3, 4] concerning the online time complexity of the perfect rainbow tradeoff with checkpoints and sometimes even the articles [3, 4] themselves will be referred to as AJO08. Similarly, references to the contents of [5] and [6] will be made with H10 and WL13, respectively.

## Comparison of AJO08 and H10

In this section, we explain how AJO08 and H10 differ in their theoretical claims and provide experiment results that closely match H10 but not AJO08.

### Theoretical Claims

The following claim of AJO08 is an almost verbatim copy of Theorem 8 from [4], with the only differences being in the characters used for the indices.

**Claim 1** (AJO08). *Given N, m, t, and a checkpoint at c, the work to rule out a false alarm when searching in column x is*

$$\begin{array}{c} Q\left(x\right)=\sum_{i=x}^{i=t}\left(i-1\right)(q_{i}-q_{c}\cdot g_{c}\left(t-i\right)), \end{array}$$

*where*

$$\begin{array}{c} q_{k}=1-\frac{m}{N}-\frac{(k-1)k}{t(t+1)}, \end{array}$$

*and*

$$\begin{array}{c} g_{c}\left(s\right)=\{\begin{array}{c} 0\;if\;there\;is\;no\;checkpoint\;in\;column\;c,\\ 0\;if\;\left(c+s\right)\leq t,\;i.e.,\;the\;chain\;generated\;from\;Y_{1}\;does\;not\;reach\;column\;c,\\ Pr\{G\left(X_{j,c}\right)\neq G\left(Y_{c+s-t}\right)|X_{j,c}\neq X_{c+s-t}\}\;otherwise. \end{array} \end{array}$$

It will not be necessary to understand what the symbols *Y*_1_, *G*(), *X*_*j*,*c*_, *X*_*j*,*c*+*s*−*t*_, and *Y*_*c*+*s*−*t*_ appearing in this claim means. The precise wording of this claim is slightly misleading, but we can infer from other parts of AJO08 that the authors had meant for the formula *Q*(*x*) to represent the cost of resolving (false) alarms incurred while searching *up to* the *x*-th column, rather than when searching *just* the *x*-th column.

No clearly marked proposition of H10 summarizes its analyses of the checkpoint technique, and the closest analogue of Claim 1 given by H10 is a statement surrounding Eq (23) of [5]. The claim reproduced below is a slightly edited version of the statement, but the content has not been altered in any way.

**Claim 2** (H10). …, *we can state*

$$\begin{array}{c} \sum_{d<k\leq t}\left(t-k+1\right){\left(1-\frac{m}{N}\right)}^{k-1}〔\frac{dm}{2N}-\frac{m^{2}k\left(k+1\right)}{8N^{2}}-\{\frac{dm}{2N}+\ln(1-\frac{dm}{2N})\}\frac{\left(k-d)(k-d+2\right)}{d^{2}}〕 \end{array}$$

*as the number of one-way function iterations that can be removed through a single 1-bit checkpoint at the* (*t* − *d*)-*th column*.

Both Claim 1 and Claim 2 concern a single pre-computation table equipped with a single checkpoint, and we will likewise restrict our discussion here to the same situation. Since Claim 2 only considers the case when the checkpoint information is set to a single bit, let us further restrict our discussion to the same case. As was stated previously, we will assume that the checkpoint is located at the *c*-th column.

The two computational complexities stated by Claim 1 and Claim 2 do not correspond to each other through any direct simple relation, and this prevents us from comparing these two results in a straightforward manner. However, one can find that the proofs of these two claims are commonly centered on the following concept.

$$\begin{array}{c} {\text{Pr}}^{\text{NFA}}\left(i\right)=〔\text{the}\;\text{probability}\;\text{for}\;\text{an}\;\text{online}\;\text{chain}\;\text{that}\;\text{starts}\;\text{from}\;\text{the}\;i\text{-th}\;\text{column}\;\text{to}\;\text{cause}\;\text{an}\;\text{alarm}\;\text{that}\;\text{escapes}\;\text{the}\;\text{filtering}\;\text{out}\;\text{process}\;\text{provided}\;\text{by}\;\text{the}\;\text{1-bit}\;\text{checkpoint}\;\text{at}\;\text{the}\;c\text{-th}\;\text{column}〕. \end{array}$$ (1)

The superscript NFA may be understood as meaning Not Filtered Alarms. For now, our interest lies only in the *i* < *c* case, since the *i* ≥ *c* case reduces to the probability of alarms when no checkpoints are in use.

The proof of Claim 1 given by AJO08 stated the alarm probability Pr^NFA^ as

$$\begin{array}{c} {\text{Pr}}_{\text{AJO}}^{\text{NFA}}\left(i\right)={\bar{q}}_{i}-\frac{1}{2}{\bar{q}}_{c}, \end{array}$$ (2)

where

$$\begin{array}{c} {\bar{q}}_{k}=1-\frac{k^{2}}{t^{2}}. \end{array}$$ (3)

Note that the new symbol ${\bar{q}}_{k}$ approximates the symbol *q*_*k*_ appearing in Claim 1, up to $1+O(\frac{1}{t})$ factor. In fact, formula ${\text{Pr}}_{\text{AJO}}^{\text{NFA}}\left(i\right)$ may be interpreted as the simplification of the term (*q*_*i*_ − *q*_*c*_ ⋅ *g*_*c*_(*t* − *i*)) appearing in *Q*(*x*) of Claim 1 for the *i* < *c* case. We clarify that even though AJO08 used *t* to denote the number of columns in the pre-computation matrix and we are using it to denote the length of the pre-computation chains, this small detail can be ignored through the $1+O(\frac{1}{t})$ factor approximation.

The first displayed equation from Section 5.2 of [5] presented the claim of H10 for the alarm probability Pr^NFA^, and when the various necessary components found within the paper are substituted, the equation becomes

$$\begin{array}{ccc} & & \frac{m(1+k)}{2N}(1-\frac{mk}{4N})-\frac{m}{N}+\frac{m(k-d+1)}{2N}\\ & & \;+\frac{\left(k-d)(k-d+2\right)}{d^{2}}\{\frac{md}{N}+2\ln\left(1-\frac{md}{2N}\right)\}. \end{array}$$ (4)

In terms of the notation used in the current paper, the above can be expressed as

$$\begin{array}{ccc} {\text{Pr}}_{\text{H}}^{\text{NFA}}\left(i\right) & = & \frac{m(t-i)}{2N}\{1-\frac{m(t-i)}{4N}\}+\frac{m(c-i)}{2N}\\ & & +\;\frac{{\left(c-i\right)}^{2}}{{\left(t-c\right)}^{2}}\{\frac{m(t-c)}{2N}+\ln(1-\frac{m(t-c)}{2N})\}, \end{array}$$ (5)

disregarding an approximation of $1+O(\frac{1}{t})$ factor.

Since the arguments of AJO08 and H10 that connected the formulas ${\text{Pr}}_{\text{AJO}}^{\text{NFA}}$ and ${\text{Pr}}_{\text{H}}^{\text{NFA}}$ to Claim 1 and Claim 2, respectively, were quite straightforward, it is reasonable to attempt a comparison of AJO08 and H10 through the probability claims ${\text{Pr}}_{\text{AJO}}^{\text{NFA}}$ and ${\text{Pr}}_{\text{H}}^{\text{NFA}}$. However, there is still one more issue that needs to be cleared before we can exercise such a comparison.

The work H10 made it explicit (in the first paragraph of [5, Section 4.2]) that they were dealing with the general perfect rainbow tables and not just the maximal perfect rainbow tables. That is, Claim 2 and Formula (5) were asserted to be valid for all perfect rainbow tables. On the other hand, even though the precise statement of Claim 1 made no restrictions concerning perfect rainbow tables, the formula it gave as *q*_*k*_ was developed in AJO08 for only the maximal perfect rainbow tables, and no explanation was given as to how this related to the non-maximal situation. Furthermore, since the term $\frac{m}{N}$ appearing in *q*_*k*_ is negligible of $O(\frac{1}{t})$ order, the formula *Q*(*x*) is essentially independent of *m*, and cannot possibly be valid for varying *m* values, when under a fixed *t*. In short, Claim 1 can only be valid for the maximal perfect rainbow tables, while Claim 2 has been stated for all perfect rainbow tables. Hence, we will restrict our comparison of AJO08 and H10 further to just the maximal perfect rainbow table case.

**Fig 1:**
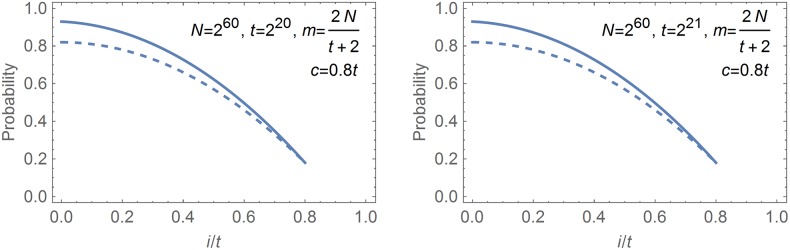
Theoretically claimed probabilities for an online chain that starts from the *i*-th column to cause an alarm that is not filtered out by a single 1-bit checkpoint. Dashed line: Claim of AJO08 given by Eq (2); Solid line: Claim of H10 given by Eq (5).

The works AJO08 and H10 agree in that the identity

$$\begin{array}{c} m=\frac{2N}{t+2} \end{array}$$ (6)

characterizes the maximal perfect rainbow tables. Graphs of the theoretically obtained formulas (2) and (5), plotted under two specific sets of parameters that satisfy the condition Eq (6), are given in Fig 1. The dashed lines represent ${\text{Pr}}_{\text{AJO}}^{\text{NFA}}$, as given by Formula (2), and the solid lines represent ${\text{Pr}}_{\text{H}}^{\text{NFA}}$, as given by Formula (5). We have somewhat arbitrarily set the checkpoint positions to *c* = 0.8*t* for the two graphs. It is clear from the graphs that ${\text{Pr}}_{\text{AJO}}^{\text{NFA}}$ and ${\text{Pr}}_{\text{H}}^{\text{NFA}}$ are fundamentally different formulas that cannot somehow be interpreted as being approximations of each other. It is clear that at least one of the two formulas must be incorrect.

### Experimental Verification of Alarm Probability

To identify which of the two claimed formulas ${\text{Pr}}_{\text{AJO}}^{\text{NFA}}$ and ${\text{Pr}}_{\text{H}}^{\text{NFA}}$, if any, represents the true probability of alarms Pr^NFA^ correctly, we conducted an experiment that measures Pr^NFA^ directly.

Our choices of *N* and *t* to use in the experiment were mostly dictated by the amount of computational resources we had available. The search space was fixed to all bit strings of 36-bit length, so that we have *N* = 2^36^. Since theoretical treatments of the rainbow tradeoff typically focus on $t\approx N^{\frac{1}{3}}$, we chose to use *t* = 2^12^. The straightforward creation of a maximal perfect rainbow table corresponding to the stated parameters requires *N* ⋅ *t* = 2^48^ iterations of the one-way function and this took us over eight days on a somewhat outdated system of 128 CPU cores.

The optimal position for a single checkpoint is indicated by Table IV of [4] to be approximately 0.89*t* and the same is claimed by Table 7 of [5] to be 0.78*t*. The difference is likely to be mostly due to the fact that Table IV of [4] considers four rainbow tables, whereas Table 7 of [5] considers a single table. Based on these information, we fixed the position of our single checkpoint to *c* = 3400 = 0.83*t*, which should be a reasonably realistic value, regardless of which of the two analyses turns out to be correct.

A slightly modified version of the MD5 hash function was used as the one-way function in our experiment. Recall that MD5 operates iteratively on 512-bit segments of its input. Since the length of our inputs was fixed to 36 bits, rather than conforming precisely to the length-related padding scheme specified for MD5, we placed the 36-bit input at the least significant end of a 512-bit block and filled the rest with zeros, before applying the usual 4-round/64-step operations of MD5. We fetched the least significant 36 bits from the 128-bit MD5 output and took it as the output of our one-way function. The reduction function was set to XOR the column number, so that the *i*-th colored one-way function XORs the integer *i* to the 36-bit output of the modified MD5.

After fully generating the *N* pre-computation chains of length *t*, we sorted them on the ending points and retained just one chain from every group of merging chains. This resulted in a pre-computation table containing *m* = 33514551 entries. This is quite close to the value $\frac{2N}{t+2}=3.3538\times 10^{7}$ predicted by Eq (6) and serves as a sanity check for our experiment. Each entry of the pre-computation table consisted of one 5-byte slot that held a 36-bit ending point value and one 1-byte slot containing the 1-bit checkpoint information. We did not keep a record of the starting points as they were not needed in identifying chain merges. The size of the resulting maximal perfect rainbow table was approximately 200 MBs and we did not apply any storage reduction techniques, such as ending point truncation or index files.

With the pre-computation table ready, the probability of alarm Pr^NFA^(*i*) could be measured experimentally. A starting column *i* was fixed and 10^6^ online chains of length (*t* − *i*) were generated from 36-bit inputs chosen at random. The number of alarms that remained not filtered even after application of the 1-bit checkpoint information were counted. We did not regenerate the pre-computation chains to verify whether any of these alarms lead to the discovery of the original input. The process of generating 10^6^ online chains was repeated for a small number of different starting columns.

The results of our experiment are summarized in the bottom row of Table 1. It is very clear that calculations made with ${\text{Pr}}_{\text{H}}^{\text{NFA}}$ of Formula (5) match the experimentally obtained figures accurately and that the predictions made with ${\text{Pr}}_{\text{AJO}}^{\text{NFA}}$ of Formula (2) do not. One can only conclude that ${\text{Pr}}_{\text{AJO}}^{\text{NFA}}$ is not an accurate formula for the alarm probability Pr^NFA^, defined by Eq (1), even for the very special case of maximal perfect tables.

**Table 1:** Probabilities for an online chain to incur an alarm that is not filtered out by a single 1-bit checkpoint.

start column *i*	0	200	400	800	1600	3200
${\text{Pr}}_{\text{AJO}}^{\text{NFA}}\left(i\right)$	0.84451	0.84213	0.83498	0.80637	0.69193	0.23416
${\text{Pr}}_{\text{H}}^{\text{NFA}}\left(i\right)$	0.94077	0.93510	0.92435	0.88767	0.75352	0.24205
test	0.94082	0.93510	0.92477	0.88798	0.75407	0.24289

The rainbow table used was maximal perfect of parameters *N* = 2^36^ and *t* = 2^12^, which resulted in *m* = 33514551. The checkpoint was placed at column *c* = 3400.

### Source of Error

Let us explain where the mistake was made by AJO08 in its formulation of the alarm probability claim ${\text{Pr}}_{\text{AJO}}^{\text{NFA}}$.

It was stated by AJO08 within the proofs of Theorem 3 and Theorem 8 of [4] that ${\bar{q}}_{i}$, as given by Eq (3), provides the probability for an online chain that starts from the *i*-th column to bring about an alarm in the absence of checkpoints. The same probability of merge was stated by H10 within the proof of Theorem 2 in [5] to be

$$\begin{array}{c} {\text{Pr}}^{\text{Mrg}}\left(i\right)=\frac{m(t-i)}{N}\{1-\frac{m(t-i)}{4N}\}, \end{array}$$ (7)

where we are, once again, ignoring a multiplicative factor of $1+O(\frac{1}{t})$ order. For the case of maximal perfect rainbow tables, we can use Eq (6) to replace $\frac{m}{N}$ with $\frac{2}{t}$, and rewrite this as

$$\begin{array}{c} \frac{2(t-i)}{t}\{1-\frac{t-i}{2t}\}=1-\frac{i^{2}}{t^{2}}, \end{array}$$ (8)

which is precisely ${\bar{q}}_{i}$. In other words, AJO08 and H10 agree on the probability of alarms in the absence of checkpoints, at least for the special case when a maximal perfect rainbow table is in use. Hence, we are lead to believe that the error hiding in Formula (2) must be in its $\frac{1}{2}{\bar{q}}_{c}$ term.

The intention of AJO08 must have been to subtract the probability for a merge to be filtered out by the checkpoint from the probability of all merges, and the use of the $\frac{1}{2}$ factor for this purpose is appropriate when dealing with a 1-bit checkpoint. The problem is with the ${\bar{q}}_{c}$ part, which AJO08 must have interpreted as the probability for the online chain to merge into the perfect pre-computation matrix after it has passed over the checkpoint column. Such a use of ${\bar{q}}_{c}$ would have been correct if the online chain under consideration started from the *c*-th column, but the use is incorrect in the current situation, because the online chain started from the *i*-th column, where *i* < *c*.

To explain the details, we need to return to the basics and treat the iteration function as a random function, as is done by any theoretical treatment of the rainbow tradeoff. An online chain that starts from the *i*-th column will loose its freedom to take its next step randomly, as soon as it merges into any one of the pre-computation chains that were generated during the pre-computation phase, including those that were discarded through the removal of ending point collisions. Hence, by the time the online chain that started from the *i*-th column arrives at the *c*-th column, one may or may not be dealing with iterations of a random function, even if the online chain had not yet merged into the *perfectize* pre-computation matrix. One simply cannot make any logical connection between the probability for a chain that starts from the *i*-th column to merge into the pre-computation matrix after passing over the *c*-th column and the probability of merge for a chain that starts afresh from the *c*-th column.

## Comparison of WL13 and H10

Test results described in the previous section showed H10 to be correct, at least when the maximal perfect rainbow tables are in use. Since H10 treated all perfect rainbow tables in a uniform manner, with the intention of having their theory applied mainly to tables that are far from maximal, validity of the theory at the extreme situation indicates that the theory is likely to be correct in the general case. The comparison of WL13 and H10 given in this section presents a natural opportunity to check the non-maximal table case more directly.

The checkpoint analyses of the perfect rainbow tables done by H10 only covered the single 1-bit checkpoint case and its extension to the multiple 1-bit checkpoints case, which H10 dismissed as being straightforward, was made explicit by WL13. However, to our surprise, the restriction of WL13 to the single 1-bit checkpoint case did not result in H10.

### Theoretical Claims

The point of divergence between WL13 and H10 in their theories was not too difficult to locate. In Section 4 of [6], WL13 wrote

$$\begin{array}{c} Pr\{F^{k-d}\left(x\right)\in RT_{t-d}\}=\frac{m(1+k-d)}{N}\{1-\frac{m(k-d)}{4N}\}, \end{array}$$ (9)

for an *x* that is chosen at random from the search space. The corresponding statement that was made by H10, i.e., Eq (21) of [5], is

$$\begin{array}{ccc} & & |F^{-(k-d)}\left({\mathrm{RM}}_{t-d}\right)|\\ & & \;=m\left(k-d+1\right)+\frac{\left(k-d)(k-d+2\right)}{d^{2}}\{md+2N\ln(1-\frac{md}{2N})\}. \end{array}$$ (10)

Instead of explaining the notation appearing in the above two equations, let us rewrite the above two claims in the notation of the present paper, assuming a single 1-bit checkpoint at the *c*-th column.

Both of the above claims concern the following probability of chain merge.

$$\begin{array}{c} {\text{Pr}}^{\text{EMr}}\left(i\right)=〔\text{the}\;\text{probability}\;\text{for}\;\text{an}\;\text{online}\;\text{chain}\;\text{that}\;\text{starts}\;\text{from}\;\text{the}\;i\text{-th}\;\text{column}\;\text{to}\;\text{have}\;\text{merged}\;\text{into}\;\text{the}\;\text{pre-computation}\;\text{matrix}\;\text{corresponding}\;\text{to}\;\text{the}\;\text{perfect}\;\text{table}\;\text{by}\;\text{the}\;\text{time}\;\text{it}\;\text{reaches}\;\text{the}\;c\text{-th}\;\text{column}〕. \end{array}$$ (11)

The superscript EMr may be understood as meaning Early Merge. We clarify that the merge of the online chain (only) into pre-computation chains that were discarded during the ending point collision removal process is not to be counted toward this probability. Statement Eq (9) from WL13 is the claim that, for *i* ≤ *c*, the probability Pr^EMr^ is

$$\begin{array}{c} {\text{Pr}}_{\text{WL}}^{\text{EMr}}\left(i\right)=\frac{m(c-i)}{N}\{1-\frac{m(c-i)}{4N}\}, \end{array}$$ (12)

while statement Eq (10) from H10 asserts that the same probability is

$$\begin{array}{c} {\text{Pr}}_{\text{H}}^{\text{EMr}}\left(i\right)=\frac{m(c-i)}{N}+\frac{{\left(c-i\right)}^{2}}{{\left(t-c\right)}^{2}}\{\frac{m(t-c)}{N}+2\ln(1-\frac{m(t-c)}{2N})\}. \end{array}$$ (13)

The reader may have noticed that the formula ${\text{Pr}}_{\text{WL}}^{\text{EMr}}\left(i\right)$ is identical to the probability of all merges Pr^Mrg^(*i*), given by Eq (7), except that every (*t* − *i*) has been replaced by (*c* − *i*), and may have accepted this as a logical claim. The implicit reasoning is that, for any fixed perfect pre-computation matrix, the probability of merge should depend only on the number of one-way function iterations taken by the online chain.

**Fig 2:**
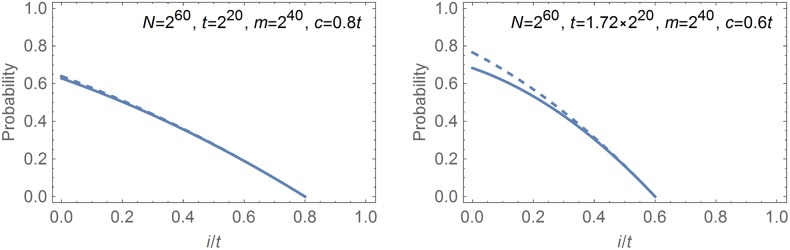
Theoretically claimed probabilities for an online chain that starts from the *i*-th column to have merged into the perfect pre-computation matrix by the time it reaches the *c*-th column. Dashed line: Claim of WL13 given by Eq (12); Solid line: Claim of H10 given by Eq (13).

Graphs of the theoretically obtained Formulas (12) and (13) for the early merge probability Pr^EMr^, plotted under two specific sets of parameters, are given in Fig 2. The dashed lines represent ${\text{Pr}}_{\text{WL}}^{\text{EMr}}$ and the solid lines represent ${\text{Pr}}_{\text{H}}^{\text{EMr}}$. The two curves appearing in the left-hand side box are very close to each other, but the two curves contained in the right-hand side box are clearly different. In view of the right-hand side box, we can state that the formulas ${\text{Pr}}_{\text{WL}}^{\text{EMr}}$ and ${\text{Pr}}_{\text{H}}^{\text{EMr}}$ are essentially different and that they cannot be seen as being approximations of each other. It is clear that at least one of the two formulas is incorrect.

We acknowledge that the parameters and the position of the checkpoint for the right-hand side box were intentionally chosen so that the two curves are easily distinguishable. However, these parameters still satisfy $\frac{mt}{N}=1.72$, so that they are reasonably practical choices. For example, to achieve a 99.9% success rate, one is most likely to use *ℓ* = 4 perfect rainbow tables with parameters satisfying $\frac{mt}{N}=-\frac{\ln(1-0.999)}{\ell }=1.72$, and the checkpoint position *c* = 0.6*t* could be under consideration when one is utilizing multiple checkpoints.

### Experimental Verification of Early Merge Probability

We conducted an experiment that measures the probability of early merge Pr^EMr^ directly, so as to determine which of the two Formulas (12) and (13) represents the true Pr^EMr^ accurately.

The slightly modified version of MD5 that was explained in the previous section was again used as our one-way function. The search space size was fixed to *N* = 2^42^ and we chose to use *t* = 20000 = 2^14.288^ and the checkpoint position *c* = 12000 = 0.6*t*. We generated *m*_0_ = 3 × 2^30^ pre-computation chains of length *t*. The starting points were not recorded, but the checkpoints and the ending points were both recorded in full. After removal of ending point collisions, we were left with *m* = 386932399 = 2^28.528^ chains. This is very close to the value $\frac{N}{N/m_{0}+t/2}=3.8697\times 10^{8}$ predicted by Eq (4) of [5]. To complete our preparation for measuring the early merge probability Pr^EMr^, we discarded all ending points and re-sorted the table according to the fully recorded checkpoints.

A starting column was fixed and online chains were generated up to the checkpoint column from 10^6^ randomly chosen 42-bit inputs. These online checkpoints were searched for in our pre-computation table of checkpoints, and the number of matches was recorded. This process of generating 10^6^ online chains up to the checkpoint column was repeated for a small number of different starting columns.

Results of our experiment are summarized in the bottom row of Table 2. The figures calculated from ${\text{Pr}}_{\text{H}}^{\text{EMr}}$ of Eq (13) match the experimentally obtained figures accurately, but those calculated from ${\text{Pr}}_{\text{WL}}^{\text{EMr}}$ of Eq (12) do not. One cannot claim ${\text{Pr}}_{\text{WL}}^{\text{EMr}}$ to be an accurate formula for the early merge probability Pr^EMr^.

**Table 2:** Probabilities for an online chain to have merged into the perfect pre-computation matrix by the time it reaches the checkpoint column.

start column *i*	0	250	500	1000	3000	6000
${\text{Pr}}_{\text{WL}}^{\text{EMr}}\left(i\right)$	0.77709	0.76659	0.75584	0.73362	0.63507	0.45821
${\text{Pr}}_{\text{H}}^{\text{EMr}}\left(i\right)$	0.68756	0.68075	0.67361	0.65839	0.58470	0.43582
test	0.68686	0.68051	0.67327	0.65862	0.58505	0.43626

Parameters: *N* = 2^42^, *t* = 20000, *m* = 3.8693 × 10^8^, *c* = 12000.

### Source of Error

The findings of the previous sub-section may have come as a surprise to the reader, if the short argument written below Eq (13) that related ${\text{Pr}}_{\text{WL}}^{\text{EMr}}$ to Pr^Mrg^ had seemed reasonable. Since Pr^Mrg^, as given by Eq (7), is the correct probability for an online chain to merge into the pre-computation matrix by the time it reaches the ending *t*-th column, replacing every *t* with *c* should *obviously* be the probability for a merge to occur by the *c*-th column. In fact, this is a straightforward application of Formula (7) to the perfect rainbow matrix of size *m* × (*c* + 1), consisting of the first (*c* + 1) columns of the full *m* × (*t* + 1) pre-computation matrix.

The fallacy in this argument lies in that it neglects certain, admittedly obscure, differences in the ending point collision removal process. The full pre-computation matrix was obtained by discarding just enough chains to remove duplicate ending points and Formula (7) is valid for this situation. However, the *m* × (*c* + 1) sub-matrix that was mentioned has lost many more pre-computation chains than was necessary to make it free of duplicates at its *c*-th column. Hence, the sub-matrix is not a naturally occurring perfect rainbow matrix, and one cannot claim that any logic is behind the tweaking of Pr^Mrg^ into ${\text{Pr}}_{\text{WL}}^{\text{EMr}}$.

**Fig 3:**
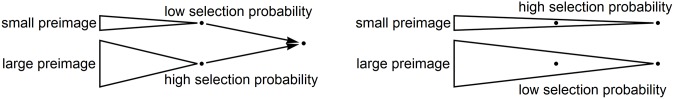
Selection of checkpoints through the selection of starting points.

We can provide a slightly more intriguing discussion. The ending point collision removal process can be viewed as the choosing of one starting point from each group of starting points that are connected to a common ending point. Using the left-hand side diagram of Fig 3 as a guide, let us mentally visualize the non-perfect pre-computation matrix before the removal of ending point collisions and consider two points on the *c*-th column that are connected to a common ending point. One might argue that, of the two points, the one that has a larger number of starting point ancestors than the other has a higher chance of remaining in the final perfect matrix. In view of random function arguments, this seems to imply that the true Pr^EMr^ would be larger than ${\text{Pr}}_{\text{WL}}^{\text{EMr}}$, but Table 2 shows that this is false. The invisible push in the opposite direction must lie elsewhere.

Note that the selection of the starting points done to remove collisions was made randomly within each group of points that are connected to the same ending point. Using the right-hand side diagram of Fig 3 as a guide, it seems plausible that the starting point ancestor sizes of the *c*-th column points would be correlated to the ancestor sizes of their corresponding ending points. Since only a single point is chosen from each group of starting points that share a common ending point, the *c*-th column points of larger ancestor sizes are chosen less often. This gives one explanation as to why the true Pr^EMr^ Would be smaller than ${\text{Pr}}_{\text{WL}}^{\text{EMr}}$.

We are not claiming that the above confusing discussion reveals the true nature of what is happening behind the scene concerning the early merge probability. However, we hope the reader is at least convinced that the previously claimed connection between Pr^EMr^ and ${\text{Pr}}_{\text{WL}}^{\text{EMr}}$, which seemed so obviously true at first, was too naive.

## Further Discussion

The previous two sections have shown that the theoretical claims concerning the checkpoint technique given by AJO08 and WL13 contained incorrect arguments and that the analysis of H10 was correct. However, since both AJO08 and WL13 provided experimental data to support their respective theoretical claims, the reader may be under the impression that either the experiments of AJO08 and WL13 were invalid or that the experiments given in the current paper has to be invalid. Let us explain that none of the experimental evidences contradict each other.

The work AJO08 provided experimental data through Fig 8 of [4], which displayed two curves corresponding to their theory and experiment. However, it can only be noticed that these two curves are visibly different at their middle parts. In particular, at the checkpoint position 7000, the *time gain* of a single 1-bit checkpoint predicted by their theory is at least 20% larger than that measured through their experiment. Hence, the experiment data provided by AJO08 is not a strong indication of the correctness of their theoretical analyses. In fact, if their experiment data are taken to be accurate, one can only conclude that their theory is quite inadequate in predicting what occurs in reality.

The work WL13 provided experimental evidence in support of their theoretical analyses through Table 3 of [6], and the similarity between their theory and experiment is quite impressive. To reconcile the apparent contradiction, we note that the parameters used in their experiment satisfy $\frac{mt}{N}=1.17$. As we saw through the left-hand side box of Fig 2, the discrepancies between the theories of WL13 and H10 can be small when parameters are such that the matrix stopping constant $\frac{mt}{N}$ is small. More precisely, if the ln(⋯)-term of ${\text{Pr}}_{\text{H}}^{\text{EMr}}$, as given by Eq (13), is replaced by just the first two terms appearing in its series expansion

$$\begin{array}{c} \ln(1-\frac{m(t-c)}{2N})=-\{\frac{m(t-c)}{2N}\}-\frac{1}{2}{\left\{\frac{m(t-c)}{2N}\right\}}^{2}-\frac{1}{3}{\left\{\frac{m(t-c)}{2N}\right\}}^{3}\cdots , \end{array}$$ (14)

we obtain ${\text{Pr}}_{\text{WL}}^{\text{EMr}}$ of Eq (12). This shows that formula ${\text{Pr}}_{\text{WL}}^{\text{EMr}}$ can serve as a reasonable approximation of ${\text{Pr}}_{\text{H}}^{\text{EMr}}$, as long as $\frac{m(t-c)}{2N}$ is small. In fact, for the specific parameters that were used in the experiments of WL13, one can confirm through graphs analogous to Fig 2 that ${\text{Pr}}_{\text{WL}}^{\text{EMr}}$ and ${\text{Pr}}_{\text{H}}^{\text{EMr}}$ are quite close to each other.

The previous two paragraph should be enough to convince the reader that the experiments of AJO08, WL13, and this work can all be valid without any contradictions, but we wish to discuss another issue that provides further understanding of the situation. In the previous sections, we focused on the probabilities Pr^NFA^ and Pr^EMr^, and we came to the conclusion that the correct formulas for these were provided by H10. We now wish to discuss how much affect the errors made by AJO08 and WL13 had on their final claims concerning the computational complexity of the complete online phase.

It is easy to check that the formulas given by Eqs (5), (7), and (13) satisfy

$$\begin{array}{c} {\text{Pr}}_{\text{H}}^{\text{NFA}}\left(i\right)={\text{Pr}}^{\text{Mrg}}\left(i\right)-\frac{1}{2}\{{\text{Pr}}^{\text{Mrg}}\left(i\right)-{\text{Pr}}_{\text{H}}^{\text{EMr}}\left(i\right)\}. \end{array}$$ (15)

Since we can expect half of the merges that occur past the checkpoint to be filtered out by the 1-bit checkpoint information, this is a logical connection of the three probability notions appearing in this equation. Similarly, restricting WL13 to the single checkpoint case, we find that the analogous claim of

$$\begin{array}{c} {\text{Pr}}_{\text{WL}}^{\text{NFA}}\left(i\right)={\text{Pr}}^{\text{Mrg}}\left(i\right)-\frac{1}{2}\{{\text{Pr}}^{\text{Mrg}}\left(i\right)-{\text{Pr}}_{\text{WL}}^{\text{EMr}}\left(i\right)\}, \end{array}$$ (16)

for the probability of alarms Pr^NFA^, is compatible with their presentation. As for AJO08, the reader may recall that we had already interpreted the corresponding claim ${\text{Pr}}_{\text{AJO}}^{\text{NFA}}$ of Eq (2) as having this structure.

The alarm probability claims we have summarized so far concerns the *i* < *c* situation. When *i* ≥ *c*, the alarm probability reduces to the case where checkpoints are not used, and the probabilities claimed by the three works can be re-obtained by simply removing the negative $\frac{1}{2}\left\{\cdots \right\}$ terms from the *i* < *c* case formulas. In other words, both WL13 and H10 would claim ${\text{Pr}}^{\text{NFA}}\left(i\right)={\text{Pr}}^{\text{Mrg}}\left(i\right)$, and AJO08 would claim ${\text{Pr}}^{\text{NFA}}\left(i\right)={\bar{q}}_{i}$, for *i* ≥ *c*.

Given Pr^NFA^, the probability for an online chain to bring about an alarm that requires the regeneration of a pre-computation chain, the number of one-way function iterations required during the online phase to treat alarms can be stated as

$$\begin{array}{c} T_{\text{NFA}}=\ell \sum_{i=0}^{t-1}i{\left(1-\frac{m}{N}\right)}^{\ell (t-i-1)}{\text{Pr}}^{\text{NFA}}\left(i\right), \end{array}$$ (17)

where we are assuming the use of *ℓ* tables and a single 1-bit checkpoint on each table. We state that this formula is compatible with the arguments of all three works AJO08, H10, and WL13. More precisely, AJO08 stated this complexity with the summations grouped in a different manner and with slightly finer granularity, while H10 and WL13 stated the reduction in time complexity brought by the checkpoints, rather than the above complexity of the work that remains. However, to the best of our understanding of the three works, they would all agreed that Formula (17) gives the time complexity associated with the alarms, as long as their respective formulas for Pr^NFA^(*i*) are used. Of course, as was done throughout this work, we are disregarding small differences that can be absorbed by an approximation of $1+O(\frac{1}{t})$ factor.

Examples of specific time complexities associated with the resolving of alarms computed with Eq (17) for the three works under consideration are given in Table 3. The complexities were computed using parameters that are typically considered during theoretical treatments of the rainbow tradeoff, under a small number of success rate requirements that would be of interest. Specifically, for each success rate requirement *p*, we used

$$\begin{array}{c} m={\left(\alpha N\right)}^{\frac{2}{3}},\;t={\left(\alpha N\right)}^{\frac{1}{3}},\;\text{and}\;\ell =\lceil -\frac{1}{2}\ln\left(1-p\right)\rceil , \end{array}$$ (18)

with the matrix stopping constant $\alpha =\frac{mt}{N}$ set to $-\frac{\ln(1-p)}{\ell }$. The position of the single 1-bit checkpoint was always fixed to *c* = 0.8*t*. All calculations were done with *N* = 2^50^, but this choice has very little effect on the values in the table, as long as *N* is not too small. The parameter sets for the slightly peculiar success rate requirements 86.46% and 98.16% correspond to pre-computation tables that are very close to being maximal perfect tables. Values given by these two rows may be the only ones that are meaningful for the AJO08 case.

**Table 3:** Various theoretically claimed online time complexities computed for specific sets of parameters.

success rate	*T*_NFA_/*t*^2^			*T*_NCA_/*t*^2^	*T*_OCG_/*t*^2^
	AJO08	H10	WL13		
99.9%	0.08829	0.07915	0.07926	0.09270	0.08316
99%	0.11219	0.09236	0.09256	0.11152	0.13353
98.16%	0.08827	0.09172	0.09215	0.11086	0.11377
90%	0.15056	0.09979	0.10000	0.12275	0.25264
86.46%	0.08391	0.08865	0.08937	0.10808	0.14854
75%	0.10593	0.08406	0.08440	0.10305	0.20992

The time complexities are those associated with the following: NFA = alarms that escape checkpoint filtering, NCA = alarms when no checkpoints are used, OCG = generation of the online chains. The parameters for each success rate requirement *p* were set to *N* = 2^50^, $\ell =\lceil -\frac{1}{2}\ln\left(1-p\right)\rceil$, $\alpha =-\frac{\ln(1-p)}{\ell }$, $m={\left(\alpha N\right)}^{\frac{2}{3}}$, $t={\left(\alpha N\right)}^{\frac{1}{3}}$, and *c* = 0.8*t*.

For easy reference, we have also included the time complexities associated with the resolving of alarms for the case when no checkpoints are used in Table 3. This was calculated with the formula

$$\begin{array}{c} T_{\text{NCA}}=\ell \sum_{i=0}^{t-1}i{\left(1-\frac{m}{N}\right)}^{\ell (t-i-1)}{\text{Pr}}^{\text{Mrg}}\left(i\right), \end{array}$$ (19)

which was stated by H10 and is compatible with the arguments of WL13. In the maximal perfect table case, the above reduces to

$$\begin{array}{c} \ell \sum_{i=0}^{t-1}i{\left(1-\frac{m}{N}\right)}^{\ell (t-i-1)}(1-\frac{i^{2}}{t^{2}}), \end{array}$$ (20)

which is compatible with the formula used by AJO08. The final column of Table 3 presents the time complexities associated with the generation of the online chains. These were calculated with the formula

$$\begin{array}{c} T_{\text{OCG}}=\ell \sum_{i=0}^{t-1}\left(t-i-1\right){\left(1-\frac{m}{N}\right)}^{\ell (t-i-1)}, \end{array}$$ (21)

which the three works can all agree on, assuming small differences that can be absorbed by approximations of $1+O(\frac{1}{t})$ factor are ignored.

Comparing the numeric values claimed by H10 for the cost of treating alarms, which is correct, with those claimed by WL13, which we now know are based on incorrect arguments, we see that the differences are quite small. Furthermore, taking note of the values provided in the *T*_OCG_ column, one finds that the differences will be even less visible when one is interested only in the total online time complexity *T*_NFA_ + *T*_OCG_. The discrepancies between the values projected by H10 and AJO08 are somewhat larger than the H10 versus WL13 case, but those rows corresponding to the use of near-maximal perfect table(s) show differences that might be acceptable for certain purposes.

The unexpected level of accuracy seen in the final time complexities that were computed using incorrect arguments can be explained. We observed through Figs 1 and 2 that the probabilities Pr^NFA^(*i*) projected by AJO08 and WL13 were most inaccurate at the starting point column. Incidentally, this happens to be where the $i{\left(1-\frac{m}{N}\right)}^{\ell (t-i-1)}$ factor appearing in Eq (17) is the smallest. Thus, the inaccuracies in the merge probabilities are dampened out when one computes the associated time complexities *T*_NFA_.

Finally, we acknowledge that the extension of H10 to multiple checkpoints elaborated on by WL13 becomes correct, if every use of ${\text{Pr}}_{\text{WL}}^{\text{EMr}}$ in their paper is replaced by ${\text{Pr}}_{\text{H}}^{\text{EMr}}$. This extension has since appeared in [7].

## Conclusion

In this work, we examined the theoretical analyses presented by AJO08 [3, 4], H10 [5], and WL13 [6] concerning the application of the checkpoint technique to the perfect table rainbow tradeoff. There were overlaps in the situations treated by the three works, and we demonstrated that their claims did not agree on the overlaps. We carried out experiments to measure the effects of the checkpoint technique on these overlapping situations. This confirmed that the arguments and claims of H10 were correct and that those of AJO08 and WL13 were erroneous. Supported by the experimental evidence, we clearly exposed where the errors were introduced in AJO08 and WL13, explaining why the associated seemingly plausible arguments were invalid.

The insight into the inner workings of the checkpoint technique gained through this work should help researchers working with iterations of the random function to avoid making similar mistakes. This work should also be of help to practitioners referencing articles on the complexity analyses of the tradeoff algorithms.
